# Pharmacological sensitivity of reflexive and nonreflexive outcomes as a correlate of the sensory and affective responses to visceral pain in mice

**DOI:** 10.1038/s41598-017-13987-9

**Published:** 2017-10-18

**Authors:** Beatriz de la Puente, Daniel Zamanillo, Luz Romero, José M. Vela, Manuel Merlos, Enrique Portillo-Salido

**Affiliations:** Drug Discovery and Preclinical Development, ESTEVE, Barcelona, Spain

## Abstract

Pain encompasses both sensory and affective dimensions which can be differentially modulated by drugs. Here, we compare the pharmacological sensitivity of the sensory and affective responses using acetic acid-induced abdominal writhings (sensory-reflexive outcome) and acetic acid-induced depression of reward seeking behaviour (RSB, affective-nonreflexive outcome) to a highly palatable food in mice. We found that the expression of RSB critically depends on factors such as sex and previous knowledge and type of the food stimulus. Intraperitoneal administration of acetic acid (iAA) produced a long-lasting (beyond the resolution of writhing behaviour) and concentration-dependent decrease on both appetitive-approach and consummatory dimensions of RSB. Ibuprofen and diclofenac were much more potent in reversing AA-induced changes in RSB: latency to eat (ED_50_ = 2 and 0.005 mg/kg, intraperinoneally, respectively) and amount consumed (ED_50_ = 11 and 0.1 mg/kg) than in AA-induced writhing (ED_50_ = 123 and 60 mg/kg). Morphine and duloxetine inhibited the writhing response (ED_50_ = 0.8 and 6 mg/kg, respectively) but not the AA-induced changes in RSB. Caffeine was ineffective in both AA-induced writhing and RSB changes. Overall, this study characterized a preclinical mouse model of hedonic deficits induced by pain that can be used to assess affective responses as well as complementary classic reflexive approaches in the evaluation of candidate analgesics.

## Introduction

Pain has many negative consequences, including changes in the affective state and the activities of daily living^1^. Current analgesics are not fully effective and the development of new ones might be hampered by the possible lack of translation from the preclinical to the clinical setting^2^. Analgesic properties of drugs have been traditionally assessed by its ability to reduce pain-induced reflexive behaviours, which reflect the sensory-discriminative component of pain. Clinical pain research, however, uses scales and verbal reports where not only pain but also physical and emotional functioning, which reflect the affective-motivational component of pain, are evaluated^3^. Thus, animal models assessing analgesic-induced recovery of normal physical and emotional functioning (i.e., pain-related non-reflexive behaviours)^2,4^ could contribute to reduce the translational gap between a laboratory discovery and a clinical therapy, and ultimately to decrease drug attrition when moving to clinic. Of course, not instead but together with traditional assessment of analgesic-induced interruption of reflex behaviours.

The study of the affective-motivational component of pain in preclinical models is complex and moves slowly, but some innovative approaches different from those based on behavioural, often evoked hypersensitivity measures have been suggested^2^. One approach is to investigate hedonically-oriented behaviours in animals with experimental pain^5–7^. These motivation-related approaches make sense in view of the interruptive consequences of both acute and chronic pain on ongoing behaviour^8,9^. From this perspective, pain interferes with daily living activities and its persistence could cause the associated stresses of depression^10^.

In general, hedonic behaviour is thought to comprise two main phases triggered by an incentive i.e. any stimulus that activates approach behaviour: the appetitive phase and the consummatory phase. Appetitive behaviours (also known as anticipatory, preparatory, approach, or seeking behaviour) are flexible, non-stereotyped responses that bring the experimental animal in physical proximity with the goal object (e.g. the reward or reinforcer), whereas consummatory behaviours constitute the final goal-consummation sequence once the motivational stimulus or goal object is reached^11^.

Visceral noxious stimulation can broadly elicit several distinct behavioural responses in rodents: (1) reflexive responses such as abdominal writhing or withdrawal to abdominal mechanical stimulation; and (2) affective-motivational responses encompassing pain-related negative emotion: conditioned place aversion^12^, changes in facial expression^13^, and suppression of some innate behaviour, such as sweet preference, wheel running or feeding^14–18^, beyond the resolution of the overt reflexive behaviours^18^. Abdominal writhings are classically measured in studies of acute pain, and involve spinal segments that receive primary afferents from visceral (thoracic) or deep somatic (hind limb-lumbosacral) nociceptors^19^. In contrast, affective responses require processing by limbic and cortical circuits in the brain where conscious and more complex processing takes place^20,21^.

The purpose of this study was double: (1) to develop a behavioural paradigm to study the affective-nonreflexive component of visceral pain, based on hedonically-oriented behaviours and taking into account both appetitive and consummatory behaviours; and (2) to find out whether pharmacological interventions were able to differentially modulate abdominal writhings (sensory-reflexive outcome) and changes in reward seeking behaviour (affective-nonreflexive outcome) induced by pain.

## Results

### Influence of neophobia and sex on RSB

**Figure 1:**
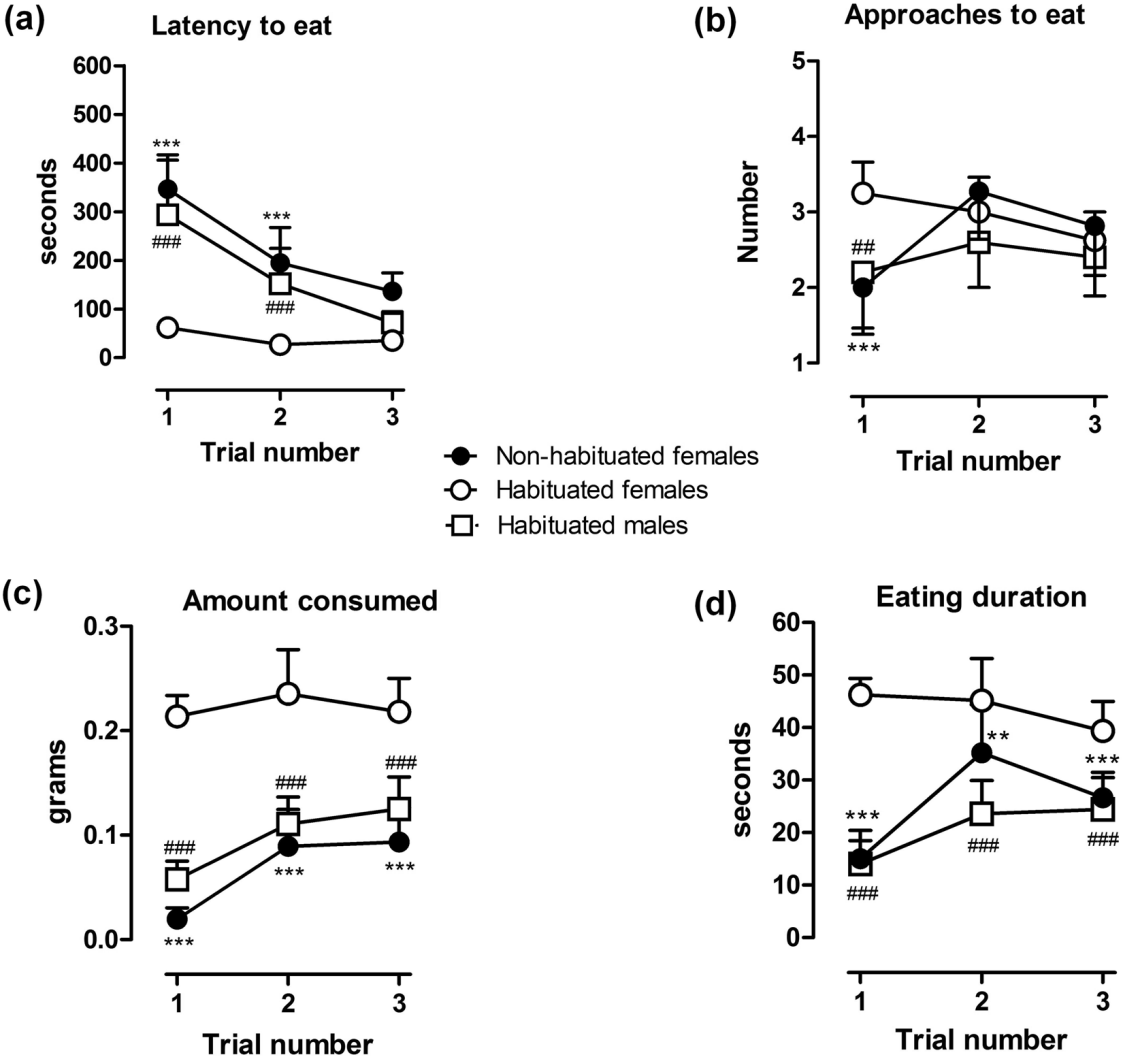
Influence of sex and neophobia on RSB. A neophobic response to white chocolate was detected by three of the four dependent measures. Habituated female mice showed a short latency to eat (**a**) as compared to both habituated male and non-habituated female mice. Amount consumed (**c**) and eating duration (**d**) were higher in female habituated than in male habituated or female non-habituated mice. Data are expressed as means ± SEM from 8 to 11 mice. Approaches to consume (**b**) were unaffected by habituation although statistical *post hoc* analysis displayed significant differences in the first trial between female habituated and female non-habituated mice. **P < 0.01, ***P < 0.001 or male habituated ^##^ *P* < 0.01, ^###^ *P* < 0.001, two-way RM ANOVA, Bonferroni’s *post hoc* test.

We first analysed the possible neophobic response to the white chocolate using female mice. A neophobic response to white chocolate was detected for three of the four dependent measures. In trial 1 the latency to eat when exposed to chocolate for the first time (non-habituated) was much higher than the latency to eat of female mice previously habituated to chocolate (Fig. 1a). In trials 2 and 3 a progressive decrease in the latency to eat was observed in non-habituated female mice, without reaching the reduced latency of habituated mice. A two-way repeated measures (RM) ANOVA test showed a significant effect of habituation condition [F_(1,51)_ = 202.6; *P* < 0.001], trial number [F_(2,51)_ = 31.0; *P* < 0.001] and a significant effect between factors [F_(2,51)_ = 17.1; *P* < 0.001]. Interestingly, no differences in the number of approaches to eat were observed between habituated and non-habituated mice [F_(1,51)_ = 3.1; *P* > 0.05] but there was a significant effect in the number of trials [F_(2,51)_ = 4.4, *P* < 0.05] or interaction between approaches to eat and the number of trials [F_(2,51)_ = 11.0, *P* < 0.001] (Fig. 1b). The amount consumed was significantly higher in female mice previously exposed to white chocolate as compared to the non-habituated group (Fig. 1c). In trial 1 the amount consumed by non-habituated mice was almost none. In trials 2 and 3 an increased consumption was observed in these mice, without reaching the consumption of habituated mice. There was a significant effect of habituation [F_(1,51)_ = 362.6; *P* < 0.001] and number of trials [F_(2,51)_ = 12.3; *P* < 0.001], and the interaction between factors was also significant [F_(2,51)_ = 6.3; *P* < 0.01]. Finally, both groups (habituated and non-habituated to white chocolate) showed a significant effect of habituation on the eating duration [F_(1,51)_ = 108.9; *P* < 0.001], trial number [F_(2,51)_ = 11.1; *P* < 0.001], or interaction between factors [F_(2,51)_ = 15.1; *P* < 0.001] (Fig. 1c). Therefore, further studies were conducted using mice habituated to white chocolate.

Second, a possible sexual dimorphism in the expression of RSB by males was analysed. We found differences between male and female mice in the four dependent measures recorded. In trial 1 the latency to eat of male mice habituated to chocolate was much higher than the latency to eat of female mice habituated to chocolate (Fig. 1a). In trials 2 and 3 a progressive decrease in the latency to eat was observed in male mice, reaching the latency of female mice. A two-way RM ANOVA test showed a significant effect of sex condition [F_(1,33)_ = 68.4; *P* < 0.001], trial number [F_(2,33)_ = 21.9; *P* < 0.001] and a significant effect between factors [F_(2,33)_ = 12.7; *P* < 0.001]. Differences in the number of approaches to eat were also observed between male and female mice [F_(1,33)_ = 11.4; *P* < 0.01], but there was no significant effect in the number of trials for each gender [F_(2,33)_ = 1.1, *P* > 0.05] or interaction between approaches to eat and the number of trials [F_(2,33)_ = 2.3, *P* > 0.05] (Fig. 1b). Furthermore, habituated male mice consumed significantly lower quantities of white chocolate and the time spent eating was also lower than habituated female mice. A two-way RM ANOVA test also showed a significant effect of sex condition [F_(1,33)_ = 157.0; *P* < 0.001] and number of trials [F_(2,33)_ = 6.1; *P* < 0.01], and the interaction between factors was also significant [F_(2,33)_ = 3.3; *P* < 0.05]. Finally, statistical analysis revealed a significant effect of sex condition on the eating duration [F_(1,33)_ = 141.9; *P* < 0.001] or interaction between factors [F_(2,33)_ = 6.9; *P* < 0.01] (Fig. 1d) but not on the trial number [F_(2,33)_ = 1.6; *P* > 0.05].

From these initial experiments we observed a clear sex difference in the approach and consumption of white chocolate, with male mice eating less white chocolate than female mice. In addition, the feeding behaviour of female mice under non-neophobic conditions was maintained high and stable in all parameters as of day two. Based on these results, the influence of pain and drugs on RSB was conducted using female mice at day 2 after the baseline determination (day 1).

### Effects of the concentration of AA on RSB

**Figure 2:**
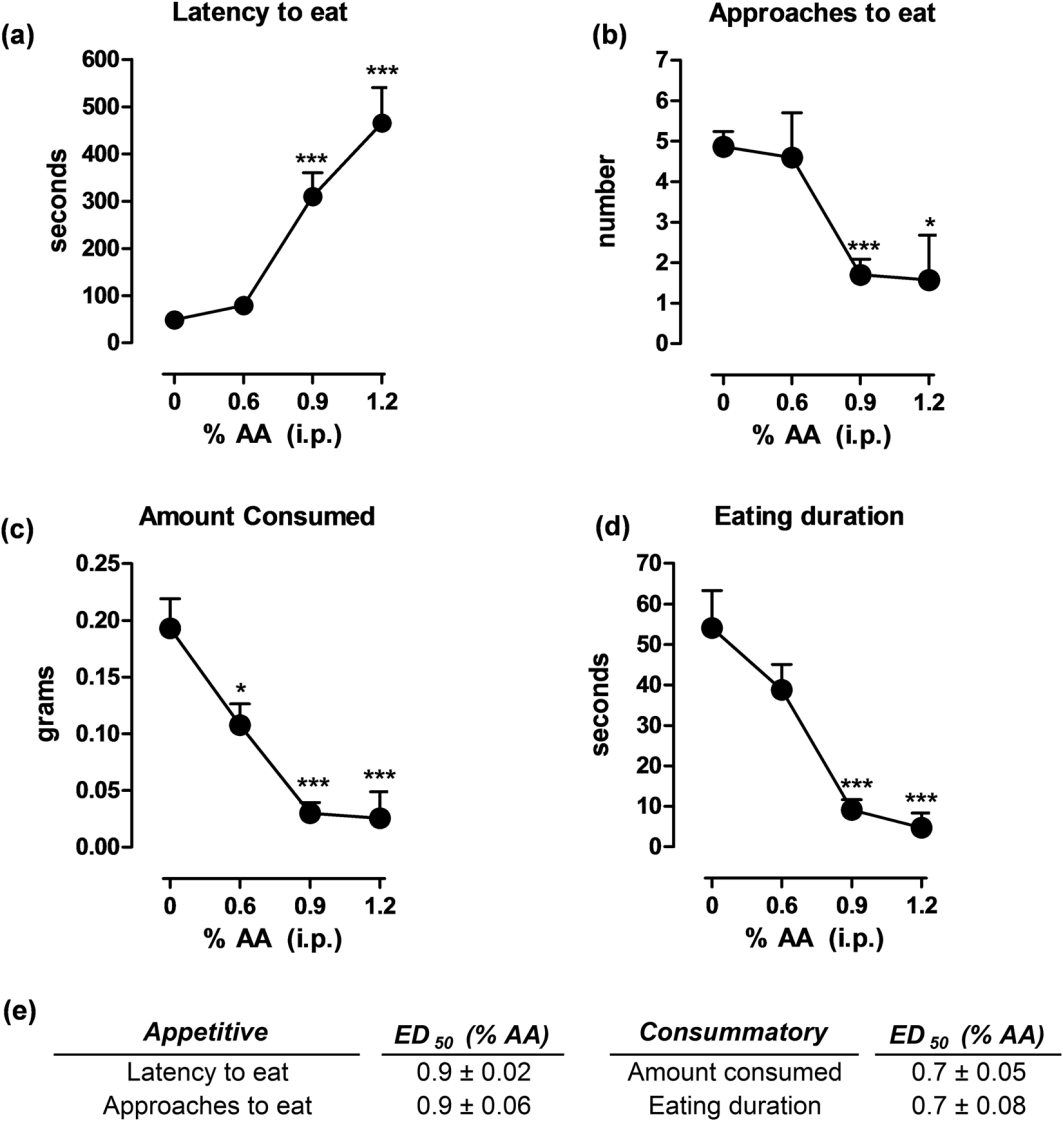
Effects of AA concentration on AA-depressed RSB. The administration of AA produced changes in a concentration-dependent manner in the four endpoints analysed. Doses of 0.9% and 1.2% AA produced significant changes in latency to eat (**a**), approaches to eat (**b**), amount consumed (**c**), and eating duration (**d**). Data are expressed as means ± SEM from 7 to 20 mice. (**e**) Values of ED_50_ for the behavioural parameters evaluated. Appetitive behaviours (latency to eat and approaches to eat) showed similar ED_50_ values (ED_50_ = 0.9%) and were higher than those obtained in consummatory behaviours (ED_50_ = 0.7%), thus suggesting that consummatory behaviours are more sensitive to changes in AA-induced pain than appetitive behaviours. *P < 0.05, ***P < 0.001, one-way ANOVA, Bonferroni’s *post hoc* test.

The RSB after i.p. injection of a range of AA concentrations in mice is shown in Fig. 2. AA administration produced changes in a concentration-dependent manner in the four endpoints analysed. Under control conditions (mice injected with HPMC and saline; the solvents of drugs and AA, respectively), the latency to eat the piece of white chocolate was 48.9 ± 9.1 seconds. 0.6% v/v AA failed to produce significant changes (79 ± 13.8; *P* > 0.05) on the latency to eat the palatable food, but 0.9% and 1.2% AA induced a strong increase (346.9 ± 39.6; *P* < 0.001; and 465.3 ± 75.1 seconds; *P* < 0.001, respectively, Fig. 2a and e for ED_50_ values). Similarly, the number of approaches to eat was not affected by 0.6% AA (from 4.9 ± 0.4 to 4.6 ± 1.1; *P* > 0.05) but it was markedly decreased by both 0.9% (1.7 ± 0.4; *P* < 0.001) and 1.2% (1.6 ± 1.1; *P* < 0.05, Fig. 2b and e for ED_50_ values) doses of AA. However, the amount of white chocolate consumed following 0.6% AA was significantly decreased (from 0.19 ± 0.03 to 0.10 ± 0.02 g; *P* < 0.05) and almost suppressed by 0.9% and 1.2% AA as compared to the control values (0.03 ± 0.009 g and 0.02 ± 0.02 g consumed, respectively; *P* < 0.001, Fig. 2c and e for ED_50_ values). Finally, doses of 0.9% and 1.2% AA, but not 0.6% AA, robustly decreased eating duration (from 54.1 ± 9.3 to 9.1 ± 2.5 and 4.7 ± 3.6 seconds, respectively; *P* < 0.001, Fig. 2d and e for ED_50_ values). In accordance to these results, further experiments were conducted using 0.9% AA, as this was the minimum concentration tested that was able to induce a robust alteration of the behaviours examined.

### Time-course of AA-induced writhing behaviour and AA-depressed RSB

**Figure 3:**
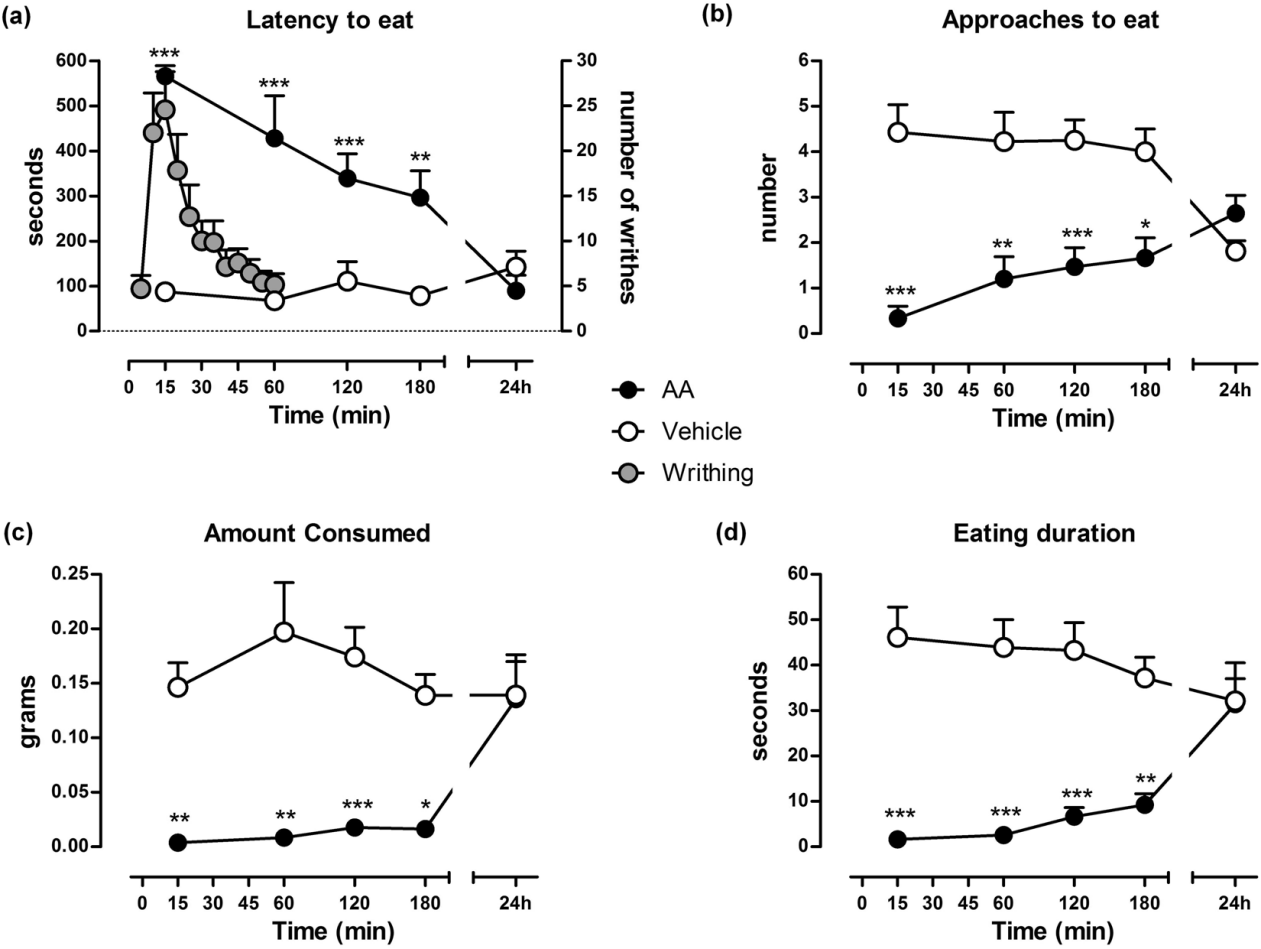
Time course of AA-induced writhing behaviour and AA-depressed RSB. Intraperitoneal administration of 0.9% AA induced writhing behaviour in mice (**a**, right axis; grey circles) than were no longer apparent after 60 min. In contrast, AA-treated mice showed RSB depression for at least 3 h. (**a**) Latency to eat, (**b**) approaches to eat, (**c**) amount consumed, (**d**) eating duration. Data are expressed as means ± SEM from 5 to 18 mice. *P < 0.05, **P < 0.01, ***P < 0.001, two-way ANOVA, Bonferroni’s *post hoc* test.

The time course of writhing behaviour and RSB following the injection of AA is shown in Fig. 3. As expected, intraperitoneal administration of 0.9% AA robustly induced abdominal constrictions (writhings) in mice (Fig. 3a, right axis and grey circles). The number of writhes was maximal from 5 to 15 minutes after AA administration (*P* < 0.001). Then, a progressive and fast decrease in this behaviour was observed, so the effects of AA were not significant after 25 min (*P* > 0.05) and no longer apparent after 60 min.

Contrary to the short duration of the writhing behaviour, AA administration produced profound changes lasting at least 180 min in the four RSB-related endpoints analysed. The left axis in Fig. 3a shows the influence of the pretreatment time of AA or its vehicle on the latency to eat white chocolate. A two-way ANOVA revealed significant effects of AA [F_(1,135)_ = 75.8; *P* < 0.001] over time after AA injection [F_(4,135)_ = 6.0; *P* < 0.001] and interaction between factors [F_(4,135)_ = 9.8; *P* < 0.001] as compared to control mice treated with the AA vehicle. The latency to eat was near the cut-off (575.4 ± 17 seconds) 15 min after AA administration and decreased slowly and progressively as of the first hour (427.8 ± 94.7 seconds; *P* < 0.001) until reaching control values 24 h later (*P* > 0.05).

The number of approaches to eat of AA-treated mice is shown in Fig. 3b. There were virtually no approaches 15 min after AA-induced pain (0.3 ± 0.2 times). A slight increase in the number of approaches (1.2 ± 0.5 times; *P* < 0.01) was observed at 1 h, and the number of approaches was similar in both AA-treated and control mice after 24 h (*P* > 0.05). While two-way ANOVA failed to indicate significant effects over time [F_(4,135)_ = 0.6; *P* > 0,05], there were significant effects of AA [F_(1,135)_ = 48.9; *P* < 0.001] and interaction between factors [F_(4,135)_ = 6.5, *P* < 0.001].

As shown in Fig. 3c, the amount consumed was highly depressed by AA administration. Two-way ANOVA indicated significant effects of AA [F_(1,135)_ = 39.6; *P* < 0.001]. No differences in the amount consumed over time [F_(4,135)_ = 1.3; *P* > 0.05] but interaction between the amount consumed and the time after AA injection [F_(4,135)_ = 2.6; *P* < 0.05] were found. Virtually there was no palatable food intake between 15 (0.004 ± 0.003 g) and 180 (0.016 ± 0.005 g) min. The amount consumed was restored to control values by 24 hours after AA-induced pain (*P* > 0.05). Similarly, there were significant effects of AA on eating duration [F_(1,135)_ = 65.0; *P* < 0.001] (Fig. 3d), but not over time [F_(4,135)_ = 0.7; *P* > 0.05]. Control mice treated with AA vehicle showed a similar eating duration at all time points analysed (*P* > 0.05 compared with 15 min). The eating duration was strongly reduced by AA at 15, 60, 120 and 180 min as compared to control values. The analysis of variance indicated that duration varied significantly between both groups [F_(4,135)_ = 4.4; *P* < 0.01]. The eating duration was restored to control values by 24 hours after AA-induced pain AA (*P* > 0.05). According to these results, subsequent RSB experiments were conducted using a 120-min pretreatment with 0.9% AA.

### Effects of morphine, ibuprofen, diclofenac, duloxetine and caffeine on AA-induced changes in the reward seeking behaviour

**Figure 4:**
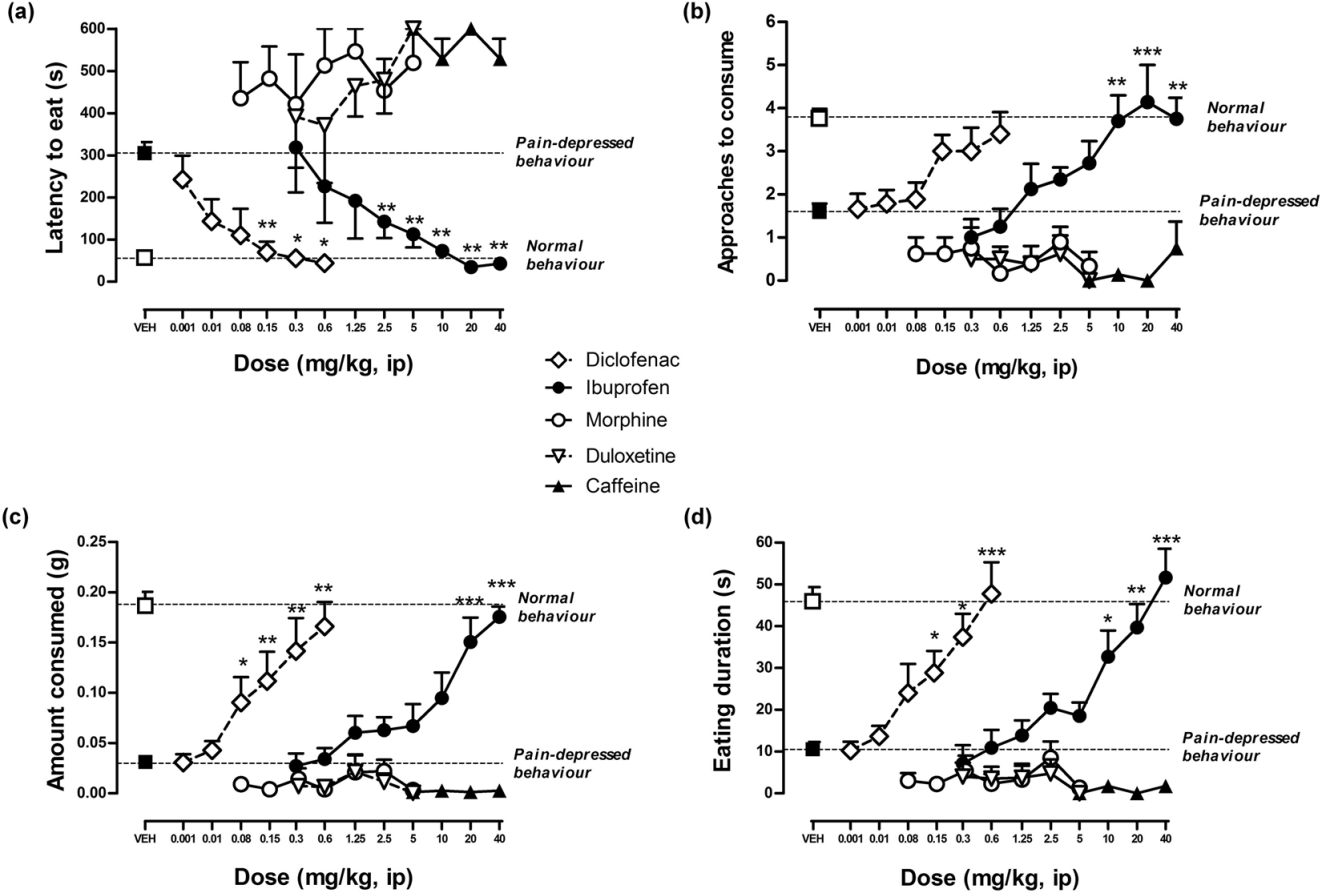
Dose response effects of diclofenac, ibuprofen, morphine, duloxetine and caffeine on the changes in RSB observed in mice with AA-induced visceral pain. Drugs were administered 90 min after AA (30 min before the test). NSAIDs drugs ibuprofen and diclofenac significantly reversed depressive-like behaviours. Morphine, duloxetine and caffeine were ineffective at any dose tested. (**a**) Latency to eat, (**b**) approaches to eat, (**c**) amount consumed and (**d**) eating duration. Data are expressed as means ± SEM from 5 to 28 mice. *P < 0.05, **P < 0.01, ***P < 0.001, one way-ANOVA, Bonferroni’s *post hoc* test.

The effects of morphine (0.08–5 mg/kg), ibuprofen (0.3–40 mg/kg), diclofenac (0.001–0.06 mg/kg), duloxetine (0.3–5 mg/kg) and caffeine (10–40 mg/kg) on the changes in RSB observed in mice with visceral pain induced by 0.9% AA are compared in Fig. 4. Treatment with the nonsteroidal anti-inflammatory drugs (NSAIDs) diclofenac and ibuprofen dose-dependently fully restored the latency to eat (Fig. 4a), the number of approaches to eat (Fig. 4b), the amount consumed (Fig. 4c), and the eating duration (Fig. 4d) of AA-treated mice to control values. Statistically significant effects at the doses of 2.5–40 mg/kg and 0.08–0.6 mg/kg for ibuprofen and diclofenac respectively were observed. However, morphine, duloxetine and caffeine were ineffective at any dose and RSB outcome tested in mice pretreated with AA.

To explore whether any of the drugs tested had effects on RSB likely to interfere in the interpretation of results, the doses of each drug tested in AA-treated mice were administered to non AA-treated mice. Table 1 shows latency to eat, duration of eating behaviour, number of approaches to eat and amount of chocolate consumed, expressed as means ± SEM for each drug and tested dose. Ibuprofen, diclofenac and caffeine did not induce significant changes in the RSB of non AA-treated mice. However, we found significant effects at the doses of 1.25–5 mg/kg and 5 mg/kg for duloxetine and morphine respectively. The latency to eat and number of approaches to eat was increased and the amount and duration was depressed by both drugs at these doses.

**Table 1:** Effects of ibuprofen, diclofenac, morphine, duloxetine and caffeine on RSB in non AA-treated mice.

	Dose (mg/kg)	Latency to eat (s)	Approaches to consume	Amount consumed (g)	Eating duration (s)
Diclofenac	**0**	75.9 ± 28.1	4.8 ± 0.9	0.1966 ± 0.04	49.4 ± 8.2
	**2**.**5**	84.3 ± 27.7	2.6 ± 0.7	0.1950 ± 0.04	33.1 ± 6.7
	**5**	73.4 ± 22.3	3.8 ± 0.7	0.1716 ± 0.03	37.0 ± 8.3
	**10**	52.1 ± 14.4	3.6 ± 0.6	0.1739 ± 0.04	40.6 ± 11.3
	**20**	106.9 ± 71.2	3.8 ± 0.8	0.1873 ± 0.04	40.6 ± 8.7
Ibuprofen	**0**	70.3 ± 13.7	4.3 ± 0.3	0.1833 ± 0.02	48.2 ± 5.2
	**2**.**5**	66.9 ± 20.1	4.6 ± 0.6	0.2043 ± 0.05	47.2 ± 11.4
	**5**	75.0 ± 19.4	4.1 ± 0.6	0.1690 ± 0.04	42.6 ± 8.0
	**10**	65.5 ± 14.1	4.6 ± 0.7	0.2010 ± 0.03	45.8 ± 5.7
	**20**	76.0 ± 17.5	4.3 ± 0.5	0.1906 ± 0.05	47.3 ± 7.9
	**40**	25.8 ± 3.2	4.1 ± 0.8	0.3387 ± 0.06	75.9 ± 13.8
Morphine	**0**	65.6 ± 18.5	4.5 ± 0.5	0.1688 ± 0.02	57.2 ± 10.1
	**0**.**3**	91.9 ± 33.3	3.7 ± 0.5	0.2651 ± 0.07*	72.7 ± 22.3
	**0**.**6**	159.0 ± 59.0	2.8 ± 0.5	0.1226 ± 0.04	37.2 ± 11.4
	**1**.**25**	105.6 ± 51.3	3.7 ± 0.7	0.1449 ± 0.02	42.5 ± 7.4
	**2**.**5**	145.1 ± 58.8	4.6 ± 1.0	0.1667 ± 0.04	40.1 ± 11.3
	**5**	278.4 ± 85.2*	2.6 ± 0.7	0.0942 ± 0.05	21.6 ± 10.0
Duloxetine	**0**	98.4 ± 37.1	2.5 ± 0.5	0.1680 ± 0.03	30.0 ± 1.0
	**1**.**25**	209.4 ± 88.0	3.1 ± 0.7	0.0814 ± 0.04	30.8 ± 11.1
	**2**.**5**	293.4 ± 84.8	1.8 ± 0.5	0.0973 ± 0.06	29.6 ± 16.0
	**5**	479.0 ± 69.6**	0.6 ± 0.3*	0.0135 ± 0.01	6.3 ± 3.8
Caffeine	**0**	52.0 ± 14.9	2.8 ± 0.3	0.1386 ± 0.04	30.5 ± 8.2
	**10**	103.5 ± 21.0	2.5 ± 0.4	0.1600 ± 0.03	32.1 ± 4.8
	**20**	42.0 ± 21.5	5.7 ± 1.1*	0.2532 ± 0.06	62.7 ± 12.8
	**40**	104.0 ± 44.2	5.3 ± 0.9*	0.2068 ± 0.06	82.3 ± 18.1**

Latency to eat (s), approaches to eat (number), amount of chocolate consumed (g) and duration of eating behaviour (s). Data are expressed as means ± SEM for each drug and tested dose from 7 to 28 mice. *P < 0.05, **P < 0.01, one way-ANOVA, Bonferroni’s *post hoc* test.

### Comparison of drugs effects on writhing (sensory-reflexive outcome) and RSB-deficit (affective-nonreflexive outcome) induced by AA

**Figure 5:**
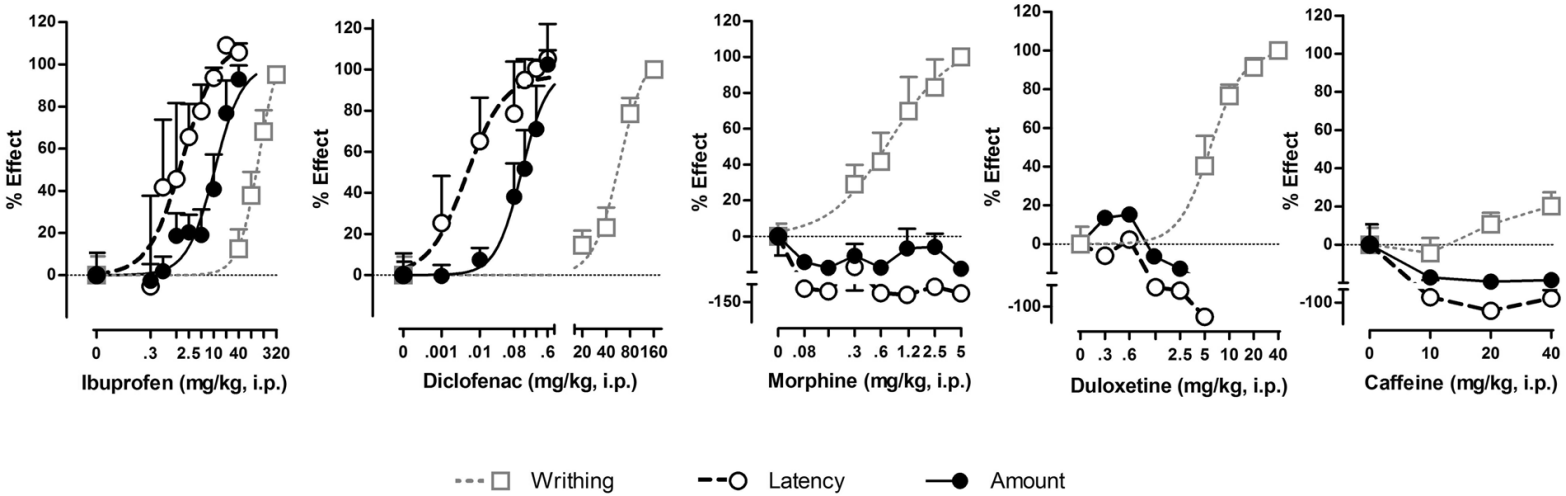
Comparison of the effect of different drugs on sensory and affective manifestations in AA-induced visceral pain. Dose response curves expressed as % of effect of ibuprofen, diclofenac, morphine, duloxetine and caffeine on two parameters of RSB (latency to eat and amount consumed) and on AA-induced writhing in mice are showed. Drugs were administered to separate groups of mice 90 min after AA to evaluate it effects on RSB changes (30 min before RSB testing) and 30 min before AA to evaluate it effects on writhing response. Ibuprofen and diclofenac displayed more potency to restore RSB (affective) than to reduce writhing behaviour (sensory-reflexive). Both drugs were also more potent in restoring latency to eat (appetitive) as compared to amount consumed (consummatory). Morphine and duloxetine blocked writhing but failed to restore RSB in mice treated with AA. Caffeine, a stimulant non-analgesic drug, lacked activity in any of the behavioural responses evaluated.

The dose response effects of morphine, ibuprofen, diclofenac, duloxetine and caffeine on two parameters of RSB and on AA-induced writhing are shown in Fig. 5a. Only the latency to eat and the amount consumed are shown as measures of the appetitive-approach and consummatory dimensions of hedonic behaviour, respectively. ED_50_ and E_max_ are shown in Table 2. Both diclofenac and ibuprofen were much more potent in inhibiting AA-induced changes in latency to eat and amount consumed than in inhibiting AA-induced writhing. Diclofenac and ibuprofen were more potent in restoring the latency to eat than in restoring the amount consumed. Finally, morphine and duloxetine inhibited AA-induced writhing in a dose-dependent manner without reversing AA-induced changes in latency to eat or amount consumed, whereas caffeine failed to show effects on any AA-induced change.

**Table 2:** ED_50_ and E_max_ values obtained in pain-depressed responses (appetitive and consumatory components of hedonic behaviour) and pain-induced reflexive responses (writhing) for all drugs evaluated.

	*Reflexive Behaviour*		*Hedonic Behaviour*			
			*Appetitive*		*Consumatory*	
	Writhing		Latency to eat		Amount consumed	
	*DE*_*50*_ ± *SE*	*E*_*max*_	*DE*_*50*_ ± *SE*	*E*_*max*_	*DE*_*50*_ ± *SE*	*E*_*max*_
Ibuprofen	122.6 ± 20.2	111.6	1.8 ± 0.8***	110.3	10.5 ± 2.0**^,###^	100
Diclofenac	59.6 ± 11.1	110.5	0.005 ± 0.004***	97.2	0.1 ± 0.03***^,#^	100
Morphine	0.8 ± 0.2	107.2	N.D.	N.D.	N.D.	N.D.
Duloxetine	5.9 ± 1.3	99.5	N.D.	N.D.	N.D.	N.D.
Caffeine	N.D.	N.D.	N.D.	N.D.	N.D.	N.D.

N.D.: not determined. *P < 0.05, **P < 0.01, ***P < 0.001, one way-ANOVA, Bonferroni’s post hoc.

## Discussion

Our first aim was to develop a behavioural model sensitive to pain revealing the affective-motivational consequences. We focused on a hedonically-oriented behaviour such as the approach and consumption of a highly palatable food whose decrease secondary to a noxious stimulus can be suggestive of a deterioration of the animal global welfare, quality of life and/or depressive-like behaviours. This behavioural test has not previously been used in pain models, but may be a useful model for research on the impact of pain on hedonic behaviour. It requires little or no training and, in addition to classic consummatory measures such as the amount consumed, a number of outcome measures such as latency and number of approaches, to separate out the concepts “wanting” and “liking”, can also be scored^22^. Furthermore, it is an ethologically relevant test that mirrors real-world experiences associated to interruption of ongoing behaviour by pain, making it more valid to the natural behaviour of a rodent than complex operant tests involving training and/or food deprivation. Since pain is often associated with depression of behaviour and mood, relief of pain-related depression is a common goal of treatment^1^. In this paradigm valuable analgesics should potentially restore the normal hedonic behaviour of the animals.

The main findings of this study were: (i) a commonly used noxious stimulus (intraperitoneal AA injection, iAA) produced a long-lasting (beyond the resolution of writhing behaviour) and concentration-dependent suppression of both the appetitive and consummatory components of hedonic behaviour in mice; (ii) this pain-related suppression of behaviour was dose-dependently prevented by treatment with ibuprofen and diclofenac but not by morphine, duloxetine or caffeine; and (iii) ibuprofen and diclofenac were much more potent in reversing iAA-induced changes in appetitive and consummatory components (affective-nonreflexive) than iAA-induced writhing (sensory-reflexive).

Methodological issues including sex, type of palatable food and neophobia were addressed in the study design. We found that male mice ate less white chocolate than female mice. This sexually dimorphic behaviour is a well-known phenomenon in rodents^23^. Neophobia to new taste is also a well described phenomenon in rodents^24^. As expected, we found that those previously receiving chocolate ate much more than those receiving standard laboratory food only. All these data indicated that the expression of sweet feeding behaviour critically depends on factors such as sex, type and previous knowledge of the food stimulus. From these experiments we adopted a strategy to optimize the expression of feeding behaviour by using non-neophobic female mice and white chocolate.

Our behavioural analysis included the recording of four related outcomes which intend to study the distinct components of motivational behaviour. Initial appetitive or motivational phase for the rewarding stimulus was inferred from changes in the latency to start the ingestion of the palatable food (free approach) and the number of approaches to eat, whereas the consumption behaviours were evaluated by amount consumed and time of consumption. Similar approaches to appetitive motivation have been used by other investigators to study motivated behaviours for natural rewards^25–27^. For instance, the time required for the mouse to traverse the alley in the “runaway” model has proven to be a reliable index of the animal’s motivation to seek the incentive that is made available to it upon goal box entry. In this paradigm, increased run time indicates a decreased motivation to reach the food reward and the degree of wanting is determined by analysing the latency to begin to consume reward during the behavioural task^28^. In our study, the latency to eat was short and stable, thus suggesting that mice were highly motivated for the incentive.

In the present study, intraperitoneal injection of a diluted solution of acetic acid was selected as a visceral noxious stimulus to induce pain. iAA is a well established animal model for tonic visceral pain in rodents (writhing test)^29–31^. iAA to rodents triggers abdominal contractions (writhing) which are a viscerosomatic reflex response to noxious irritation and are considered a manifestation of pain^30^. It is well established that the parietal peritoneum receives somatic innervation. iAA directly activates both visceral and somatic nociceptors that innervate the peritoneum and induces inflammation that is not limited to the sub-diaphragmatic organ but also to the muscle wall^30,31^. iAA suppress not only feeding behaviour but also nesting^32^, locomotor activity^15,18^ and wheel running behaviour^16^. The iAA-induced changes on these behaviours have been always attributed to the pain sensation.

Now we report that iAA administration caused a dose-dependent increase in the latency to eat, suggesting that mice appetitive motivation was affected by pain. Interestingly, our AA dose response study found that while the latency to eat the reward was not significantly affected by the dose of 0.6% AA, the amount of reward consumed was reduced by approximately 50%. This result suggests that the approach-related parameters of hedonic behaviour were more resistant than consummatory parameters to acute visceral pain. This could make sense from an evolutionary perspective since strong impediments in approach motivation —which occur before the consummatory-related behaviours— could seriously affect survival.

Our results showed that 0.9% AA induced a transient behavioural depression characterized by a strong reduction in the reward-seeking behaviour which lasted at least 3 hours and reverted to baseline levels 24 hours after injection. However, the temporal pattern of the writhing behaviour was of short duration; in fact it disappeared after one hour of the injection which suggests that the resolution of writhing reflex does not imply the resolution of pain. This offset in the recovery time of RSB was similar to a previous result obtained in the saccharine preference test, where the preference for this sweet solution was depressed for 6 hours^18^. It has been already described that the consumption of palatable food can be decreased upon exposure to AA^14^. The present study incorporated measures not only of the final consummatory phase but also of the motivational dimension of behaviour by recording the latency to eat and the number of approaches to eat.

It is well known that patients with functional gastrointestinal disorders have disproportionately high levels of psychological comorbidities such as anxiety and depression^1^. The amygdala is well known to play a pivotal role in anxiety, fear processing and depression; as well as descending inhibitory effects on nociceptive processing^33,34^. Noxious stimulation of the gut activates amygdalar neurons via vagal C-fiber input to the nucleus of the solitary tract^35^. Thus iAA could activate, among others, gut-brain pathways responsible for emotional responses, affecting the expression of RSB.

The second goal of this study was to compare drug sensitivity between affective-nonreflexive (changes in RSB) and sensory-reflexive (writhing) outcomes. Our study evaluated the pharmacological modulation of RSB two hours later, when the writhing behaviour was absent. We found that NSAIDs ibuprofen and diclofenac dose-dependently restored the RSB deficit induced by AA to control values. Ibuprofen fully restored both appetitive and consummatory aspects of RSB at much lower doses than those required to inhibit the writhing response. In fact, the restorative effect on RSB was observed at doses failing to show significant effects on writhing. Therefore, as far as the decrease in RSB reflects the affective component and the increase in writhing behaviour reflects the sensory component of AA-induced pain, ibuprofen was able to reduce the affective component of pain at doses that did not affect the sensory component of pain. Similar results were observed for diclofenac, another NSAID, which also dose-dependently restored all parameters evaluated. Diclofenac was at least 100-fold more potent than ibuprofen. Although diclofenac and ibuprofen have similar mechanism of action the potency of cyclooxygenase activity inhibition varies between both agents which could explain these results. The antinociceptive action of NSAIDs is primarily due to the inhibition of prostaglandin biosynthesis through the inhibition of cyclooxygenase enzymes: COX-1 (constitutive) and COX-2 (inducible in inflammatory processes). Diclofenac is approximately 25 fold more potent than ibuprofen to inhibit prostaglandin synthesis^36,37^. In contrast to ibuprofen, which is a nonselective NSAID, diclofenac shows preference for COX-2 inhibition over COX-1 inhibition (260 nM and 10 nM, for COX-1 and COX-2 respectively)^38^. This is particularly relevant when using low doses as reported in our study for diclofenac. The selectivity of a particular COX inhibitor is critically dependent on its concentration and diclofenac can also inhibit COX-1 at higher concentrations^39,40^. In our study, the effects of drugs on the reflexive behaviour (writhing) were studied 5 min after the iAA while the effects of drugs on the non-reflexive behaviour (approach and consumption of white chocolate) were studied 2 hours after the iAA, when writhing was over. Thus our result suggests that biochemical mechanisms underlining both behaviours are different. COX-1 expression seems to be more important for the behavioural expression of writhing and, in consequence, higher doses of diclofenac were required to inhibit COX-1 and suppress the writhing behaviour. The importance of the COX-1 in the writhing response to iAA has been confirmed in homozygous COX-1 gene deleted mice in which abdominal contractions were almost completely abolished^41^. On the other hand, the surprisingly low ED50 for diclofenac to restore the latency to eat (0.005 mg/kg) suggests that COX-2 expression is extremely relevant at the time we analyzed the drug’s effects, 2 hours after iAA. The AUC after intraperitoneal administration of diclofenac (25 mg/kg) in mice has been calculated as 232 μM × h^42^ and assuming a linear pharmacokinetics for diclofenac which has been reported^43^ we can estimate an AUC of 46 nM × h for the ED50 0.005 mg/kg, which is higher than the IC50 for the COX-2 inhibition (10 nM)^38^. Thus despite the low doses necessary to restore the appetitive behaviour in AA-treated mice, it seems feasible that diclofenac could in fact be elicit a selective inhibition of the COX-2 enzyme *in vivo* at such a low doses/exposure. Globally, our results with diclofenac are in accordance with the notion that COX-1-derived prostanoids may be generated in the initial phase of acute inflammation, while COX-2 up-regulation which requires some hours is presumably the dominant pathway in later phases of the inflammatory response^44^.

Diclofenac was also effective in restoring the affective component of pain at much lower doses than those required to restore the sensory component of pain. In addition, both drugs were more potent in restoring the motivational aspect of the hedonic response than in restoring the consummatory aspect of it, thus suggesting that approach motivation was more sensitive to analgesic drugs than consummatory pleasure. Moreover, ibuprofen and diclofenac at doses producing anti-anhedonic responses in mice with AA-induced pain had no effect on control mice administered with AA vehicle. This suggests that efficacy is not due to non-specific increases in the hedonic response as a result of NSAIDs administration.

Morphine and duloxetine dose-dependently decreased the number of writhes resulting from intraperitoneal injection of AA. These results are consistent with previous studies that have shown similar results with both drugs in assays of acid-stimulated writhing in rats and mice^14,45–47^. However, both duloxetine and morphine failed to restore any parameter evaluated in the RSB task at any dose tested, including those that produced an inhibitory effect in the assay of writhing behaviour. This result would suggest that these drugs are effective only on the sensory-related changes associated to visceral pain. Human and animal studies have shown that μ opioid agonists attenuate the affective component of pain more potently than its sensory component^2,48^. However, most of these studies have evaluated the effects of morphine on non-reflexive outcomes using models of peripherally-induced nerve injuries and using cutaneous stimulation to evaluate paw withdrawal^2^, and not visceral pain. Furthermore, the well-known gastrointestinal side effects, including nausea and vomiting^49^ and the important central rewarding effects associated to opioids^50^ may interfere, making the natural reward in our experimental conditions less attractive. In fact, previous studies using the same visceral stimulus have showed both effect and no effect of morphine to attenuate the suppression of feeding^14,16^. On the other hand, duloxetine markedly reduces food intake in rats^51^ and humans^52^. Thus, possible confounding factors associated with stimulus and pain characteristics (visceral *vs*. non-visceral), as well as the side effects of morphine and duloxetine can limit the power of our feeding-based outcome to effectively dissociate the sensory and affective pain dimensions from visceral origin. Testing drug’s effects on natural behaviours in control animal helps to interpret results by discarding possible interferences in the target behaviour, as observed with some doses of morphine and duloxetine in this study.

Finally, as expected, the non-analgesic stimulant caffeine had no significant effects on any pain-elicited and pain-depressed behavioural responses measured, at any dose tested, thus suggesting that despite the fact that caffeine normalized locomotor activity of AA-treated mice to the level of non-caffeine-treated control animals^18^, the pain-induced deficit in RSB was insensitive to the behavioural arousal induced by caffeine. iAA could potentially activate vagal afferents that regulate food intake and satiety which could contribute to the depression of feeding behavior. However, the fact that some analgesics but not the non-analgesic stimulant caffeine (negative control) restored the RSB suggests that the suppression in the RSB is triggered by the iAA-induced pain/aversiveness.

In summary, a reward seeking behaviour paradigm was developed where not only consumption but also appetitive, goal directed behaviour have been reliably disrupted by pain and sensitive to NSAIDS but not to morphine, duloxetine and caffeine. The iAA-induced deficit on RSB behaviour was probably due to ongoing pain because it was specifically reverted by an analgesic drug such as ibuprofen and diclofenac but not by the stimulant drug caffeine. NSAIDs were much more potent to restore pain-related depression of RSB than pain-related increase in writhing, thus suggesting that the affective-motivational response was more sensitive than the sensory-discriminative response to pain. However, as a limitation, analgesic side effects such as rewarding properties or food intake impairment would interfere with positive results in this paradigm. In a biopsychosocial approach to pain, measures beyond simple reflexive behaviours are mandatory to comprehensively validate and investigate painful conditions in rodents. Interruption of over behaviour by pain and its recovery by drugs can be an important area for future investigation to improve the predictive validity of preclinical studies on candidate analgesics. The extraordinarily sensitivity to some analgesics point to RSB as a valuable primary outcome measure to complement the more traditional procedures used to assess analgesics.

## Material and Methods

### Animals

All animal research was conducted in the PRBB PCB Animal Facility in accordance with protocols approved by the local Committee of Animal Use and Care of our Institution, with the Care and Use of Laboratory Animals Guidelines of the European Community (European Directive 2010/63/EU), and with the International Association for the Study of Pain Guidelines on ethical standards for investigation in animals^53^. Animal studies are reported in compliance with the ARRIVE guidelines^54,55^. The number of animals and intensity of noxious stimuli used were the minimum necessary to demonstrate consistent effects of the treatments used. Experimenters were blinded to the drug treatment when performing tests. No animals were excluded from the analysis. Each experiment was repeated two to three times (using three or four animals in each repetition) between 08:00 and 14:00 h. All tests were conducted during the light phase. Animals were euthanized immediately after the experiment by CO_2_ inhalation in an appropriate room by personal from the animal facility. Female and male CD1 mice aged 4–5 weeks were purchased from Charles River (France) and randomly housed in groups of five in transparent polypropylene cages with suitable beddings (wood shavings) and food and water *ad libitum*. Animals were left undisturbed for two weeks to acclimatize to laboratory conditions in a testing room with light and temperature maintained at 20–22 °C with a 12 h light/12 h dark cycle (lights on at 06:00 and off at 18:00).

### Drugs and compounds

Ibuprofen and duloxetine were synthesized by Laboratorios Esteve. Caffeine and diclofenac were purchased from Sigma Chemical Co. (Barcelona, Spain). Morphine hydrochloride was obtained from the Spanish Drug Agency (Agencia Española de Medicamentos y Productos Sanitarios, Area Estupefacientes, Madrid, Spain). All compounds were dissolved in 0.5% hydroxypropyl methylcellulose (HPMC) and administered intraperitoneally at a volume of 10 ml/kg. Drugs were administered 30 min before the test. AA solutions were prepared by adding 0.06, 0.09 or 0.12 ml of glacial acetic acid to double deionized water at a final volume of 10 ml. The injection volume of AA was 10 ml/kg, intraperitoneal (i.p.).

### Assay of reward seeking behaviour (RSB): approach and consumption of white chocolate (affective outcome)

The basic test procedure was adapted from Merali *et al*.^56^ who showed that offering highly palatable, familiar snacks in the home cage resulted in a rapid approach and consumption of food. In this study, among the different food sources offered to nondeprived mice, white chocolate (Milkybar®, Nestlé, S.A.) was chosen based on preliminary experiments indicating a stronger consumption as compared to other rewarding stimuli, such as dark chocolate (Supreme Mini-Treats™, Bio-Serv Inc). Each mouse was placed in one corner of the home cage (floor size 25 × 50 cm) on the opposite side from a Petri dish (4-cm-diameter, 1-cm-deep) containing a piece of white chocolate (~2 g). The animal was tested for 10 min (cut-off point) and, in order to obtain a more detailed profile of pain-induced alterations of the feeding behaviour, a total of four dependent measures were used: latency to eat, number of approaches to eat, amount of chocolate consumed and duration of eating behaviour. Latency to eat was defined as the time between the placement of the animal in the home cage containing the chocolate and the beginning of eating. The number of times and the time taken to eat chocolate were chosen because they have been shown to be affected by several experimental manipulations^57,58^. The amount consumed was assessed by weighing the piece of chocolate before and after testing.

We first studied the stability of RSB and the influence of sex and neophobia (i.e. reduction in consumption of a novel tastant because of fear to the unknown post-ingestive consequences)^59^. After the acclimatization period to the experimental room, mice were divided into three groups: Male (group 1) and female (group 2) mice habituated to white chocolate by adding a dish with 4 g of white chocolate to the home cage for three consecutive days (non-neophobic), and female mice not habituated (group 3, neophobic). Then, mice were tested, as described above, for three consecutive days (trials 1 to 3).

Second, the concentration (0–1.2% v/v) and the pretreatment time (0–24 h) of AA were systematically manipulated in order to identify conditions under which AA reliably depressed the reward-seeking behaviour. RSB was evaluated in different groups of mice at 15, 60, 120 and 180 min. A group of mice tested at 120 min was again tested at 24 h. Third, the effects of a range of doses of i.p. morphine (0.08–5 mg/kg), ibuprofen (0.3–40 mg/kg), diclofenac (0.001–0.6 mg/kg), duloxetine (0.3–5 mg/kg) and caffeine (10–40 mg/kg) were examined on AA-depressed and non-depressed RSB (vehicle treated condition). Drugs were injected 30 min before the RSB test which was conducted 2 hours after the administration of AA, when writhing behaviour have disappeared.

### Assay of AA-Induced Writhing (sensory outcome)

In the time course study, mice were injected i.p. with 10 ml/kg of AA (0.9%). Each mouse was then placed in an individual clear plastic observation chamber and the total number of writhes was counted for 1 h after administration. Based on the results obtained, a 5-min pretreatment time was used for the remainder of the writhing experiment. To evaluate the effects of drugs, separate groups of mice were administered vehicle or a dose of the corresponding drug by i.p. route followed by an intraperitoneal injection of 0.9% AA 30 min later. The number of writhes was counted from 5 to 15 min after AA administration (35–45 min after vehicle or drug). For scoring purposes, a “writhe” was defined as a contraction of the abdominal muscles accompanied by elongation of the body and extension of the hind limbs. In this assay, the effects of a range of doses of intraperitoneal (i.p.) morphine (0.3–5 mg/kg), ibuprofen (40–320 mg/kg), diclofenac (20–160 mg/kg), duloxetine (5–40 mg/kg) and caffeine (10–40 mg/kg) were examined.

### Statistical Analysis

Data are expressed as mean ± SEM. For the purpose of pretreatment time of AA, data were analysed with a two-way analysis of variance (ANOVA); the effects of sex and white chocolate habituation studies were analysed with a two-way repeated measures ANOVA. In the time course study, writhing data were analysed using a one-way repeated measures ANOVA. AA concentration data and the drug treatment study were analysed with one-way ANOVA. Bonferroni’s *post hoc* analyses were performed in all cases to assess specific group comparisons. *P* < 0.05 was considered to be statistically significant.

A dose–response curve was plotted using nonlinear regression analysis, and ED_50_ (dose of drug producing half of its maximal response) and E_max_ (maximum effect) values were obtained. Standard error (SE) were calculated on the basis of the best-fit values ± SE of regression with GraphPad Prism software (version 5.0; GraphPad Software Inc., La Jolla, California, USA).

To calculate the ED_50_, data were converted to the percentage of analgesia based on the following formula for the writhing test: [mean no. of writhes (control group) − (mean no. of writhes (test group)]/[mean no. of writhes (control group)] × 100 and the following calculation for behavioural parameters: ([mean value test group − mean value pain-AA group]/[mean value control group − mean value pain-AA group]) × 100.
